# The Scribble Polarity Protein Stabilizes E-Cadherin/p120-Catenin Binding and Blocks Retrieval of E-Cadherin to the Golgi

**DOI:** 10.1371/journal.pone.0051130

**Published:** 2012-11-30

**Authors:** Madhura Lohia, Yi Qin, Ian G. Macara

**Affiliations:** 1 Department of Microbiology, Ctr for Cell Signaling, University of Virginia School of Medicine Charlottesville, Virginia, United States of America; 2 Department of Cell and Developmental Biology, Vanderbilt University Medical Center, Nashville, United States of America; Northwestern University Feinberg School of Medicine, United States of America

## Abstract

Several polarity proteins, including Scribble (Scrb) have been implicated in control of vesicle traffic, and in particular the endocytosis of E-cadherin, but through unknown mechanisms. We now show that depletion of Scrb enhances endocytosis of E-cadherin by weakening the E-cadherin-p120catenin interaction. Unexpectedly, however, the internalized E-cadherin is not degraded but accumulates in the Golgi apparatus. Silencing p120-catenin causes degradation of E-cadherin in lysosomes, but degradation is blocked by the co-depletion of Scrb, which diverts the internalized E-cadherin to the Golgi. Loss of Scrb also enhances E-cadherin binding to retromer components, and retromer is required for Golgi accumulation of Scrb, and E-cadherin stability. These data identify a novel and unanticipated function for Scrb in blocking retromer-mediated diversion of E-cadherin to the Golgi. They provide evidence that polarity proteins can modify the intracellular itinerary for endocytosed membrane proteins.

## Introduction

Cell polarity is intimately related to vesicle sorting, which ensures the delivery to and removal from the appropriate membrane proteins to the correct territories on the cell surface, and maintains the distinct properties and functions of these territories. The machineries involved in polarization and vesicle traffic are each understood in molecular detail, but our knowledge of their interactions with one another, and their cross-regulation, remains rudimentary. The PAR proteins, plus several other polarity proteins including Lgl and the Cdc42 GTPase, have been implicated in control both of apical protein traffic and E-cadherin endocytosis from adherens junctions while, in a reciprocal fashion, vesicle sorting proteins, including several Rabs, are required for normal localization of the polarity proteins [1]–[4]. Although Par3 can bind to the exocyst complex [5], and Lgl associates with syntaxins [6], the underlying mechanisms for the interplay between these two machineries remain unclear. Scrb is required for clustering of synaptic vesicles at synapses [7], [8] and over-expressed Scrb, through a β-PIX:GIT1:ARF6 pathway, inhibits endocytosis of the thyroid stimulating hormone receptor (TSHR) and promotes recycling back to the plasma membrane [9].

Scribble (Scrb) was discovered as an apicobasal polarity protein and tumor suppressor in *Drosophila* embryonic epithelia [10], [11], but in mammals has been also implicated in planar cell polarity (PCP) [12]. Scrb contains an N-terminal LRR (Leucin Rich Repeat) domain, which is necessary for association with the lateral cortex [13], [14], and 4 PDZ domains, through which it can interact with β-Pix, a guanine nucleotide exchange factor for the Rac GTPase [15], and Vangl2, a PCP protein [16], amongst other partners. In MDCK epithelial cells grown in culture, Scrb is required for normal intercellular adhesion [17]. When these cells are depleted of Scrb, they appear more fibroblastic. They spread over a larger surface area and cortical actin rings are replaced by numerous stress fibers [17]. These effects are independent of β-Pix or of changes in Rac-GTP. Importantly, MDCK cells lacking Scrb are unable to attach to a surface coated with the extracellular domain of E-cadherin, suggesting that Scrb is required to promote E-cadherin-mediated cell-cell adhesion [17].

The homophilic connections between cadherin molecules on adjacent cells are weak; however, clustering of cadherin molecules on the lateral surface of cells strengthens cell-cell adhesion [18]. Clustering is mediated by proteins that bind to intracellular regions of the adhesion molecules and by those that affect cytoskeletal architecture. They include the armadillo repeat-containing proteins β-catenin, γ-catenin (plakoglobin), and p120-catenin (p120), which directly bind to E-cadherin and promote its junctional localization and adhesive properties [19]–[21]. Association with p120 blocks endocytosis of E-cadherin and its degradation in the lysosome [22]. The mechanisms that determine alternate fates of internalized E-cadherin remain unclear. After recruitment into early endosomes the protein can be recycled back to the plasma membrane or delivered via late endosomes to the lysosomes for degradation. The sorting nexin, SNX1 appears to promote E-cadherin recycling after stimulation of cells with EGF [23], while p120 is essential for its plasma membrane retention and to prevent degradation [22]. Sorting nexins play several roles in cargo sorting, but in particular are required for retromer-mediated retrieval of cargo to the Golgi [24], [25].

In this study we address the mechanisms by which Scrb promotes E-cadherin adhesive activity. We discovered that Scrb stabilizes the p120catenin-E-cadherin coupling to reduce the rate of E-cadherin internalization and, unexpectedly, blocks retromer-mediated retrieval of E-cadherin to the Golgi, thus implicating Scrb in the control of vesicle sorting.

**Figure 1:**
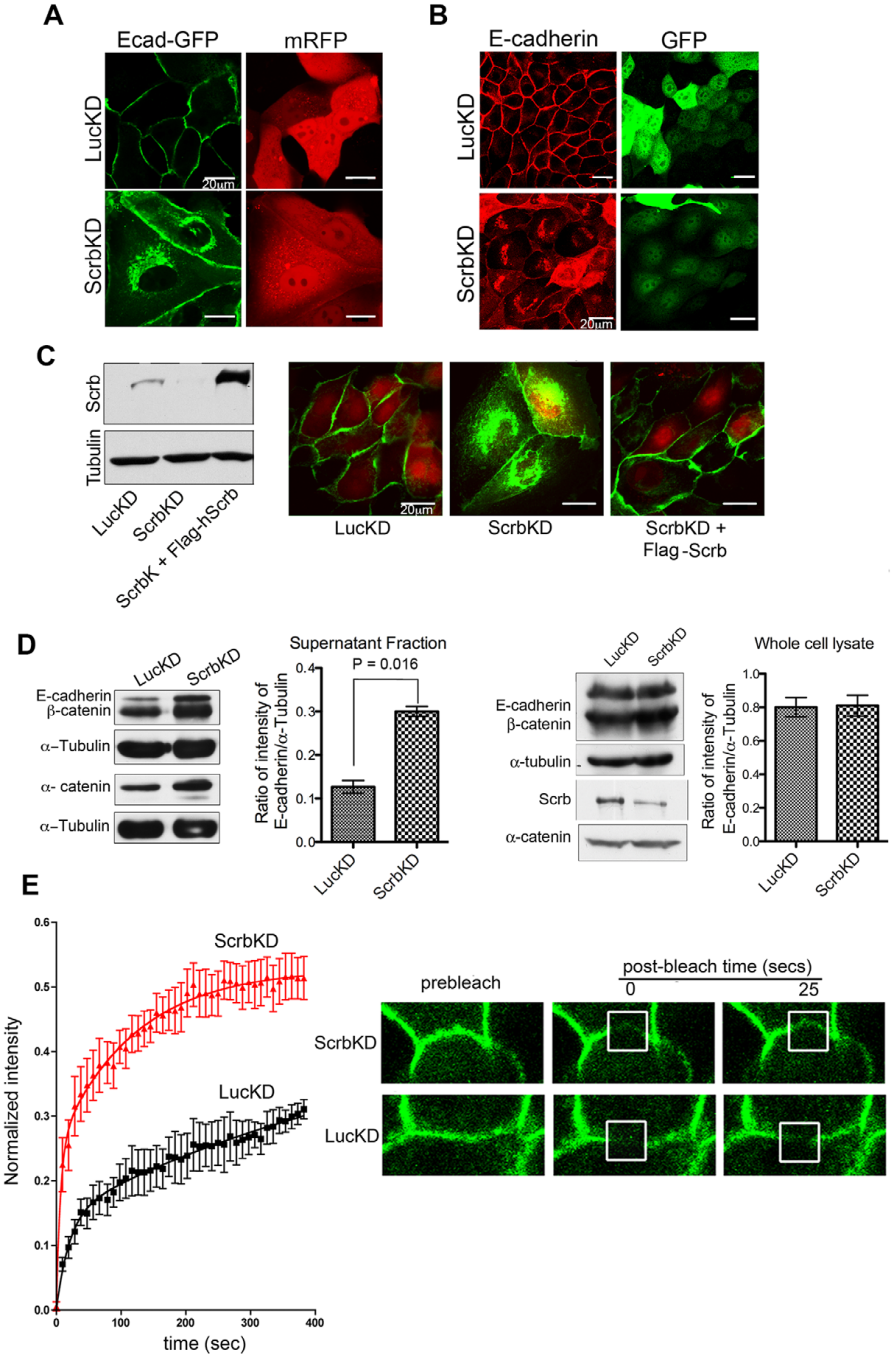
Loss of Scrb causes E-cadherin accumulation into perinuclear vesicles. (A) MDCK cells stably expressing E-cadherin-GFP (Ecad-GFP) were nucleofected with either a control shRNA (LucKD) as a control or with an shRNA that specifically targets canine Scrb (ScrbKD), along with mRFP to mark transfected cells. Cells were plated on chambered coverslips and visualized live. Scale bars are 20 µm. (B) MDCK cells were nucleofected as in A, but using GFP as a transfection marker, then plated on slides for 2 d before fixing and staining for E-cadherin and GFP. (C) Normal localization of Ecad-GFP to the lateral membranes is rescued by expression of human, Flag-tagged Scrb with shRNA against the canine Scrb. (D) Cells were lysed in hypotonic buffer with no detergent, and centrifuged at 12,000×g for 10 min. Supernatant fractions were analyzed by immunoblot for adherens junction components. Tubulin was used as a loading control. The amounts of E-cadherin in the supernatant fractions were quantified and normalized to the intensities of the α-tubulin bands. Error bars represent mean +/− SEM (n = 3). (E) FRAP analysis of E-cadherin dynamics in GFP-E-cadherin MDCK stable cell line. Confocal sections of representative cells are shown before and after photobleaching. Curves show fluorescence recovery in control and knockdown cells (n = 5) (mean +/−1 SD). Mean intensities were adjusted for bleaching during imaging, normalized by subtraction of the residual fluorescence immediately after bleaching (∼35%) and analyzed using Prism software to fit a double-exponential model for recovery. Fluorescence intensity, I = A*(1-exp(−K_1_*t))+B*(1−K_2_*t)), where t = time after bleaching; K_1_ and K_2_ are rate constants, and A and B are the maximal fractional recoveries of Ecad-GFP in each component. For control cells, regression coefficient R^2^ = 0.55, for K_1_ = 0.05+/−0.025 sec^−1^, K_2_ = 0.0018+/−0.0034 sec^−1^, A = 0.15+/−0.039, and B = 0.31+/−0.37. For Scrb-depleted cells, R^2^ = 0.68, for K_1_ = 0.158+/−0.074 sec^−1^, K_2_ = 0.008+/−0.002 sec^−1^, A = 0.25+/−0.036, and B = 0.28+/−0.03. Equal amounts of whole cell lysates from control and ScrbKD MDCK II cells were analyzed by immunoblot for Scrb expression.

## Materials and Methods

### Cell Lines and Constructs

MDCK T23 cells were obtained from Keith Mostov (UCSF, San Francisco, CA) [26]. The Ecad-GFP cell line was obtained from James Nelson (Stanford University, CA) [27]. Scrb vectors were described previously [17]. Scrb fragment constructs were generated by PCR and cloned into BamHI/EcoRI sites of pKGFP and pGEX vectors. To generate shRNAs against the canine p120catenin and Vps29, small interfering RNA primers were synthesized using rational design criteria as described previously [17]. Sequences of the sense p120catenin and Vps29 oligonucleotides are as follows:

p120KD: 5′GATCCCCGCCAGAGGTAGTTCGGATATTCAAGAGATATCCGAACTACCTCTGGCTTTTTGGAAA;

Vps29KD1: 5′ GATCCCCGAGGAGACTTCGATGAGAATTCAAGAGATTCTCATCGAAGTCTCCTCTTTTTGGAAA;

Vps29KD2: 5′- GATCCCCGAATTATCCAGAACAGAAATTCAAGAGATTTCTGTTCTGGATAATTCTTTTTGGAAA.

**Figure 2:**
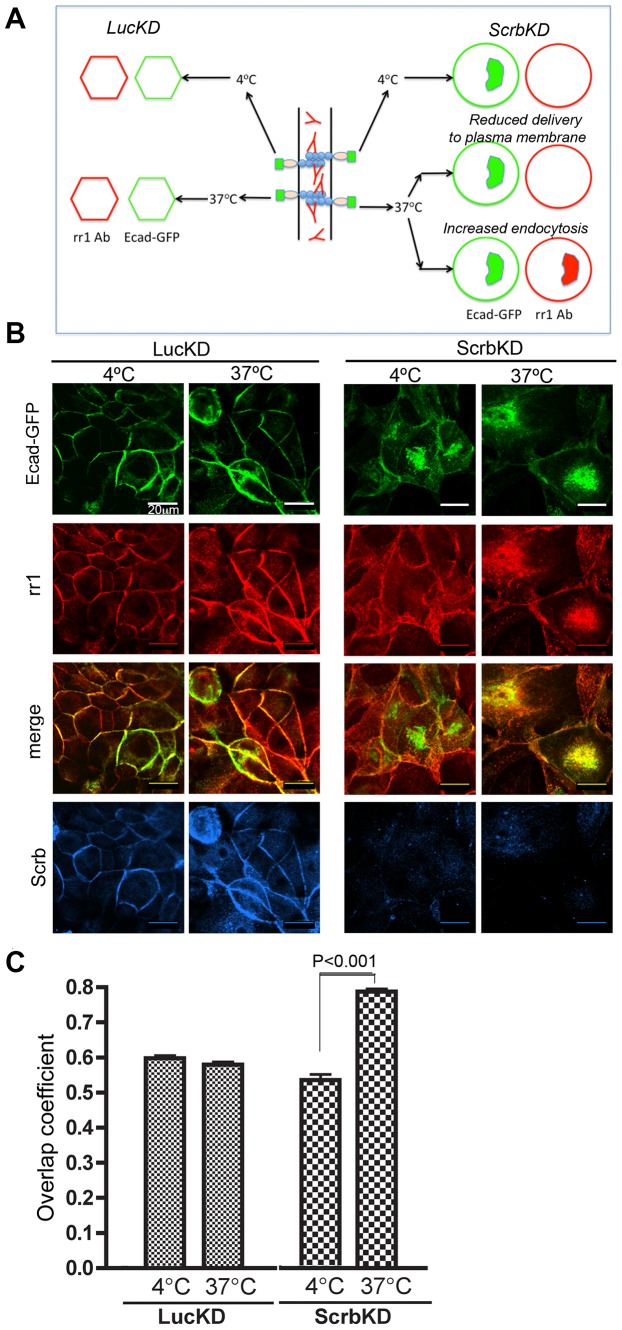
E-cadherin endocytosis is promoted in the absence of Scrb. (A) Schematic of experimental protocol to distinguish effects of Scrb depletion on delivery of E-cadherin to the plasma membrane versus on internalization of E-cadherin from the membrane. The rr1 antibody, which recognizes the extracellular domain of E-cadherin, will be retained at the plasma membrane at 4°C. Internalization is slow in control cells, so most of the antibody will still be at the plasma membrane after 2 h at 37°C. However, if internalization is accelerated by loss of Scrb, the rr1 antibody will be recruited along with E-cadherin into intracellular vesicles. (B) Control and Scrb-depleted Ecad-GFP cells were plated on 0.4 µm filters for 18 h. The E-cadherin extracellular domain-specific antibody (rr1) was added to the bottom wells and allowed to bind to cells for 1 h at 4°C. Cells were then washed to remove unbound antibody, incubated at either 4°C or 37°C for 2 h, then fixed and stained for the antibody (red) and for Scrb (blue). All images are confocal sections (Zeiss LSM 510; 40× oil immersion lens, NA 1.4). Scale bars are 20 µm. (C) Quantification of the overlap coefficient for colocalization of Ecad-GFP and rr1 (mean +/−1 SEM; n = 5 fields).

Sense and antisense oligonucleotides for shRNAs were purchased from IDT Technologies, annealed, phosphorylated, and ligated into the BglII and HindIII sites of pSUPER [28].

**Figure 3:**
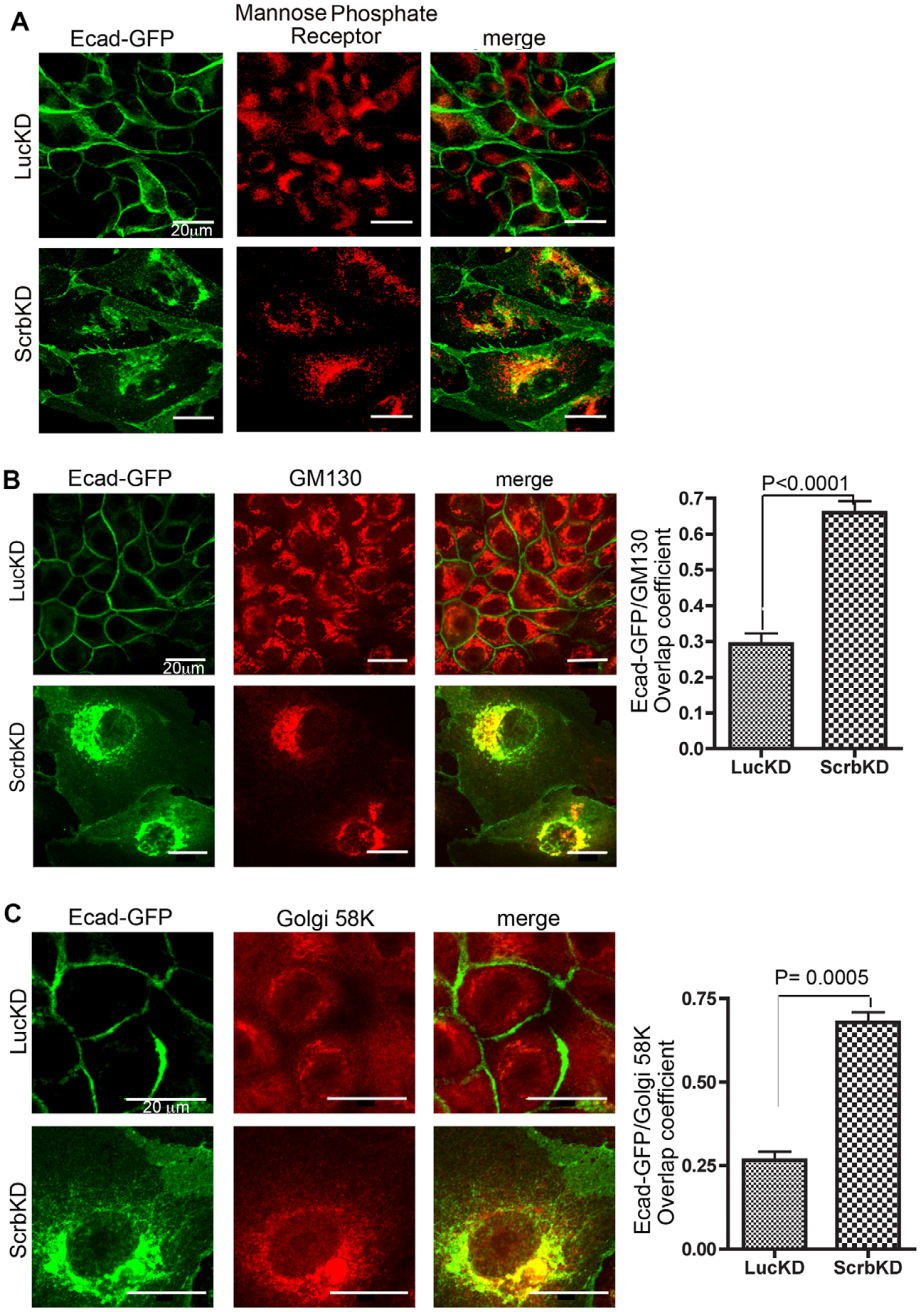
Internalized E-cadherin is retrieve to the Golgi in Scrb-depleted cells, is associated with retromer components, and requires retromer for Golgi accumulation. (A) Staining of Ecad-GFP expressing MDCK cells for mannose-6 phosphate receptor. Scale bars are 20 µm. (B) Co-localization of Ecad-GFP with the Golgi marker GM130. All images are confocal sections (Zeiss LSM 510; 40× oil immersion lens, NA 1.4). Overlap coefficients were determined using Openlab software (+/−1 SEM; n = 3). (C) Co-localization of Ecad-GFP with Golgi 58K. Overlap coefficients were determined using Openlab software (+/−1 SEM; n = 3).

### Immunological Methods

For analysis of total cell extracts by immunoblot, cells were scraped directly into Laemmli sample buffer. After SDS-PAGE, proteins were transferred to nitrocellulose and detected by chemiluminescence. For immunoblots of supernatant fractions, cells were washed with cold PBS and lysed in a detergent-free lysis buffer (50 mM Hepes, pH 7.4, 100 mM NaCl, 5 mM MgCl_2_, 0.5 mM EDTA, 10 µg/ml leupeptin, 10 µg/ml aprotinin, 1 µg/ml DNase I, 1 mM DTT, and 1 mM PMSF). After 3 rapid freeze/thaw cycles, samples were cleared by centrifugation (20 min at 14,000 rpm). Supernatants were assayed for protein concentration using Bradford reagent then boiled in sample buffer. Antibodies used were as follows: anti-Scrb (1∶100), anti-Rap1 (1∶200), anti–β-catenin (1∶1,000), anti–E-cadherin (1∶1,000), anti-p120catenin (1∶1000; BD Biosciences); anti–α-tubulin (1∶2,500; Sigma-Aldrich); anti-GFP (1∶1000; Invitrogen); anti-GST (1∶5000;). HRP-conjugated secondary antibodies were used at a dilution of 1∶5,000–1∶10,000 (Jackson ImmunoResearch Laboratories). Band intensities were quantified using ImageJ.

**Figure 4:**
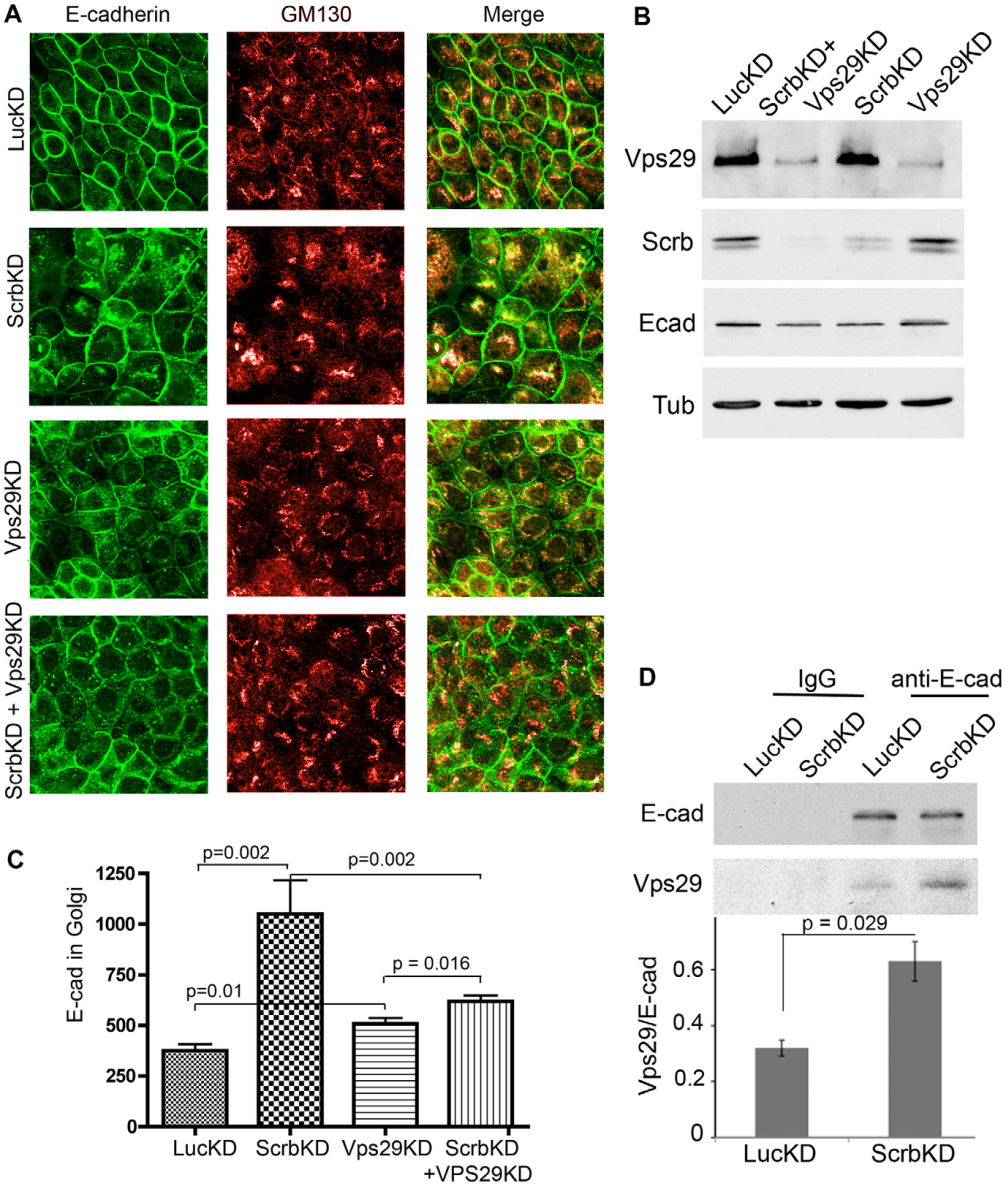
(A) Depletion of retromer component Vps29 blocks accumulation of E-cadherin in the Golgi. Ecad-GFP cells were nucleofected with shRNAs to silence Scrb and/or Vps29. After 3 d cells were fixed, permeabilized and stained for GM130 and GFP. A second shRNA against Vps29 gave identical results. Confocal images were all captured using the same channel settings. (B) Regions of interest were drawn around the Golgi area for individual cells and the mean pixel intensities were measured for GFP fluorescence. Histogram shows mean GFP fluorescence +/−1 SEM (n = 5). (C) Silencing of Scrb increases the association of E-cadherin with retromer component Vps29. Cells were nucleofected with shRNAs. After 48 h cell lysates were immunoprecipitated with anti-E-cadherin and blotted for associated Vps29. Band intensities were quantified using Image J (+/−1 SEM, n = 3).

For immunofluorescence, cells were fixed in 4% paraformaldehyde, permeabilized with 0.5% Triton X-100 in PBS, and blocked with 1% gelatin in PBS for 1 h before incubation with antibodies. For detection of Na/K-ATPase and E-cadherin, cells were permeabilized in methanol at −20°C for 10 min. Primary antibodies against Scrb (1∶50), p120catenin (1∶500), α**-**catenin (1∶500), E-cadherin (1∶500) β-catenin (1∶500), and GM130 (1∶500) were from BD Biosciences. Antibodies against Numb (1∶25) and Mannose 6-Phosphate receptor (1∶500) were from Abcam. Anti-Rab11a (1∶400) was from Invitrogen and anti-Afadin (1∶1000) and anti-Golgi 58K (1∶100) were from Sigma-Aldrich). Anti-Na/K ATPase (1∶250) was a gift from M. Caplan (Yale University, New Haven, CT). Alexa Fluor–conjugated secondary antibodies (Invitrogen) were used at a dilution of 1∶1,000. Alexa 546–conjugated phalloidin (Invitrogen) was used at a dilution of 1∶400; DRAQ5 (Cell Signaling) was used at a dilution of 1∶1000. Cells were mounted in Fluormount-G (SouthernBiotech). Lysotracker Red DND-99 was from Invitrogen.

**Figure 5:**
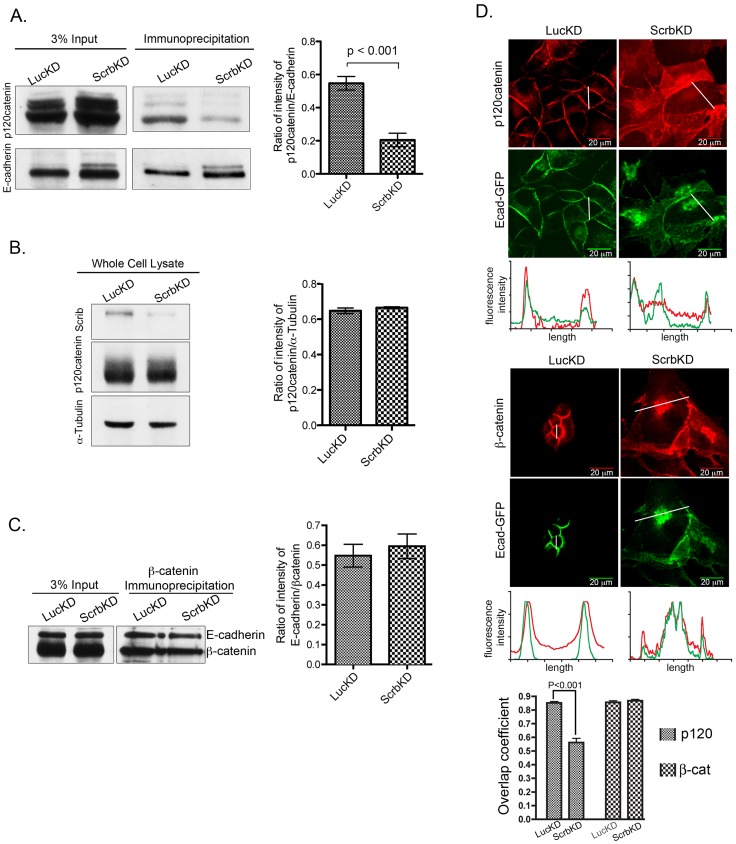
The E-cadherin/p120 interaction is disrupted in Scrb-depleted cells. (A) E-cadherin was immunoprecipitated from control and ScrbKD lysates as described in Methods, and the samples were blotted for p120. Band intensities were measured as described in Methods. Error bars = mean +/−1 SEM (n = 4). (B) Equal amounts of LucKD and ScrbKD total cell lysates were blotted for p120. Error bars indicate mean +/− SEM (n = 3). (C) β-catenin was immunoprecipitated from control and ScrbKD MDCK cell lysates and the samples were blotted for E-cadherin (mouse antibody) and β-catenin (rabbit antibody). Error bars indicate mean +/− SEM (n = 3). (D) Control and Scrb-depleted cells stably expressing Ecad-GFP were grown on slides for 48 h, then fixed and immunostained for p120 or β-catenin. Scale bars are 20 µm. Pixel intensities were measured across the lines shown and are displayed beneath the images. Quantification of co-localization between Ecad-GFP and the catenins was performed for multiple fields using Openlab. Error bars show mean +/−SEM (n = 5 fields per condition).

For immunoprecipitation experiments, cells were lysed in 50 mM Tris pH 7.5, 0.5% NP-40, 200 mM NaCl, 5 mM MgCl_2_, 1 mM DTT, 1 mM PMSF, 10 µg/ml leupeptin, 10 µg/ml aprotinin, 2 mM Na_3_VO_4_ and1 µg/ml DNase I. In some experiments lysis was performed in a hypotonic buffer lacking detergent and salt. After passing through a sub-Q 261/2 needle, lysates were centrifuged for 15 min at 10,000×g. Supernatants were immunoprecipitated for 2 h using the indicated antibodies and sheep anti-mouse IgG or sheep anti-rabbit IgG conjugated Dynabeads (Invitrogen). Samples were washed 4× in lysis buffer and once in PBS then eluted in sample buffer.

**Figure 6:**
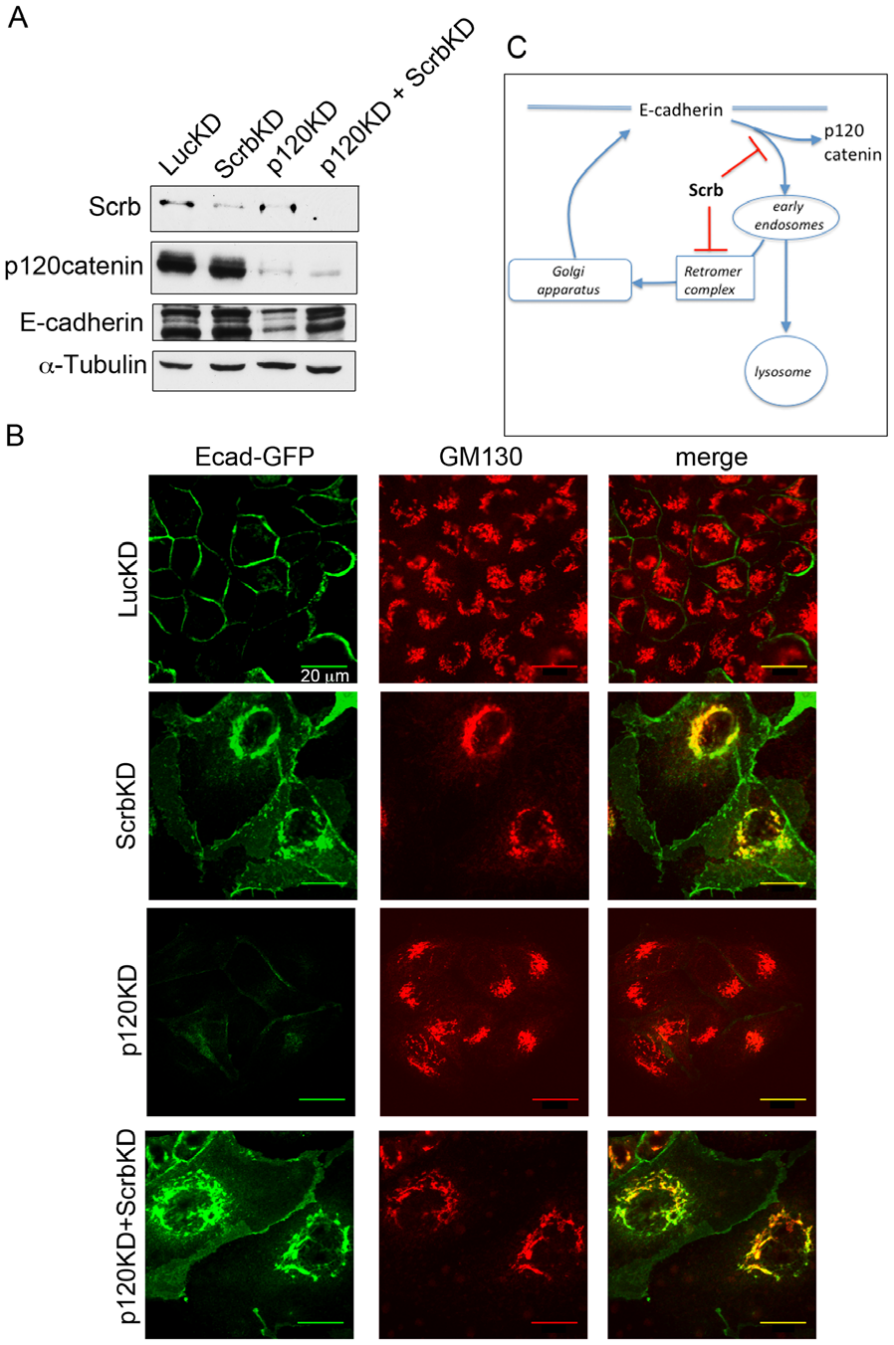
Diversion of E-cadherin to the Golgi is independent of p120. (A) Silencing Scrb reverses the loss of E-cadherin caused by depletion of p120. MDCK cells were transfected with shRNAs as shown, and incubated for 3d. Lysates were blotted for Scrb, p120 and endogenous E-cadherin. Tubulin was used as a loading control. Note the rescue of E-cadherin expression in the p120/Scrb double knockdown, as compared to the p120 single knockdown. (B) Silencing of Scrb diverts E-cadherin to the Golgi in cells lacking p120. Silencing of p120 alone results in the internalization and destruction of E-cadherin. Cells were stained for GM130 as a Golgi marker. Scale bars are 20 µm. (C) Schematic for Scrb function in regulating E-cadherin localization and recycling. Scrb is proposed to have two separate functions, first to stabilize p120 association with E-cadherin, and second to block retromer association with internalized E-cadherin, preventing its diversion to the Golgi.

### Imaging and Fluorescence Recovery after Photobleaching (FRAP)

Confocal images were captured using an LSM510 Meta microscope (Carl Zeiss, Thornwood, NY), using a 40× (1.4 NA) or 100× (1.3 NA) oil immersion lens. Phase-contrast images were captured on a TE200 widefield microscope (Nikon, Tokyo, Japan) using a 10× lens and an Orca charge-coupled device camera (Hamamatsu, Bridgewater, NJ). Images were converted to TIFF format using ImageJ and processed using the Adobe Photoshop CS4 Levels tool (Adobe Systems, Mountain View, CA) to enhance contrast.

For FRAP assays, Ecad-GFP cells [27] were transfected with either LucKD or ScrbKD plasmids. After 48 h, cells were subjected to photobleaching and imaged by confocal microscopy (Meta LSM510; Carl Zeiss), using a 100× oil immersion lens (1.3 NA). Cells were imaged with a 488-nm laser line at 75% power, 5% transmission. For photobleaching, regions of interest (ROIs) were selected between two adjacent transfected cells and bleached with the 488-nm laser line, at 75% power, 100% transmission for 3 iterations. Recovery within the ROI was monitored for 400 s. For each sample, images were recorded prebleach, and at various times postbleach. For all datapoints, mean intensities were adjusted for bleaching during imaging, normalized by subtraction of the residual fluorescence immediately after bleaching (∼35%) and analyzed using Prism software to fit a double-exponential model for recovery.

### Protein Expression and Rap Activity Assays

GST–Scrb fragments and GST-Ral1GDS were prepared as described previously [29]. Assays were performed as described previously using a GST fusion of the Rap1 binding domain of Ral1 to capture Rap1-GTP [30].

### E-cadherin Internalization Assay

LucKD or ScrbKD transfected cells were seeded onto 0.4 µm filters for 24 h. After culturing cells in serum-free medium for 1 h, medium containing anti-E-cadherin ectodomain antibody- rr1 (University of Iowa, Hybridoma Bank) at a concentration of 10 µg/ml was added to the bottom chamber of the filter plates. Cells were incubated with antibody for 1 h at 4°C and then rinsed thoroughly to remove unbound antibody. Treated cells were then incubated in culture media at either 4°C or 37°C for 3 h, fixed, permeabilized, and stained with an anti-mouse Alexa 546 secondary to detect the rr1 antibody.

## Results

### Scrb Depletion Causes an Accumulation of E-cadherin in Perinuclear Vesicles

Previously, we had shown that Scrb depletion disrupts lateral membrane organization, but had not detected significant internalization of E-cadherin [17]. However, direct visualization of Ecad-GFP showed that loss of Scrb causes a substantial fraction of the protein to accumulate into intracellular vesicles, clustered in the perinuclear region of the cell (Fig. 1A). Ecad-GFP is fully functional and localizes correctly to adherens junctions [27]. The use of a methanol/acetone mixture to fix cells can lead to the disruption of vesicular structures, so we repeated our immunostaining for endogenous E-cadherin after fixation with paraformaldehyde, followed by permeabilization with methanol. Under these conditions, internalization of the E-cadherin into intracellular vesicles can clearly be seen in cells depleted of Scrb (Fig. 1B). Note that, as we showed previously [17], loss of Scrb causes the cells to flatten and become more fibroblastic in appearance so that, when imaged en face, they appear much larger than the control epithelial cells (which have vertical lateral membranes), and a greater percentage of the E-cadherin is in focus in a confocal z-section. Importantly, the expression of Flag-tagged human Scrb, which is not targeted by the shRNA against canine Scrb, was capable of fully rescuing the cortical localization of GFP-Ecad, demonstrating that the effect on internalization of E-cadherin is specifically caused by loss of Scrb, rather than off-target responses (Fig. 1C).

As a biochemical test for vesicular redistribution of E-cadherin, MDCK cells expressing the LucKD or ScrbKD shRNAs were ruptured in hypotonic lysis buffer, and centrifuged to separate cytoskeleton-associated structures, nuclei, and other large organelles, from smaller vesicles. The supernatants were analyzed by immunoblot. Although loss of Scrb does not cause any change in total E-cadherin levels (Fig. 1D right panel, and [17]) the band intensity was significantly higher in Scrb-depleted supernatants as compared to the control (Fig. 1D, left panel), consistent with a shift of E-cadherin, and its associated proteins α- and β-catenin, into small vesicles. To assess the effect of silencing Scrb on E-cadherin dynamics at the cell membrane, we used FRAP on the Ecad-GFP cell line. Junctional regions between adjacent cells were selected for bleaching, where pixels were unsaturated (white box, Fig. 1E), and average intensity was monitored during recovery. The initial bleach reduced the fluorescence intensity by ∼67%. Recovery was characterized by a rapid recovery phase (T1/2 ∼ 60s) followed by a much slower recovery. The recovery curves could not be described using a single exponential model, but were well-fit to a double exponential model in which the rate constant for the rapid phase was >3× higher for cells depleted of Scrb (Fig. 1E), while there was no significant difference in the rate constants for the slow phase of recovery. The rapid initial phase was also ∼2× larger for Scrb-depleted cells than the control. The dynamics of the initial phase are too fast to represent delivery of protein from intracellular vesicles, since delivery of newly synthesized E-cadherin occurs on a timescale of hours [31]. Most likely, therefore, this phase corresponds to lateral diffusion of the E-cadherin within the plasma membrane.

To determine whether Scrb depletion causes a general defect in membrane protein traffic, we examined the localization of another lateral membrane-associated protein, the Na/K ATPase. As expected, in control cells Na/K ATPase was associated exclusively with the lateral cell boundaries, but the protein was also on the lateral membranes in ScrbKD cells, and was not detectable in intracellular vesicles (Fig. S1A). We conclude that Scrb is required specifically for the stabilization of E-cadherin at the cell cortex.

### Loss of Scrb Promotes Endocytosis of E-cadherin

We next asked if the intracellular accumulation of E-cadherin in Scrb-depleted cells is caused by a defect in the delivery of the protein to the surface or because of an increase in the rate of endocytosis. To distinguish these possibilities, cells expressing Ecad-GFP were transfected with Luc or Scrb shRNAs then incubated at 4°C with an antibody specific to the extracellular domains of the canine E-cadherin (rr1) [32]. After washing out unbound antibody, the cells were further incubated at either 4°C (restrictive temperature), or at 37°C (permissive temperature) for 2 h to allow endocytosis to take place. Cells were then fixed to examine the distribution of Ecad-GFP and the rr1 ectodomain antibody (Fig. 2A). In control cells, most of the antibody remained at the cell surface (Fig. 2B). In Scrb-depleted cells maintained at 4°C, at which temperature endocytosis is blocked, the antibody was also predominantly at the cell cortex, even though internalized Ecad-GFP was visible – either from new synthesis or as a result of endocytosis prior to rr1 addition. However, in Scrb-depleted cells that had been switched to 37°C for 2 h, the antibody was recruited efficiently to the perinuclear region where Ecad-GFP had accumulated (Fig. 2B) as reflected by the significant increase in the overlap coefficient (Fig. 2C). These data suggest that loss of Scrb promotes endocytosis of E-cadherin, and that E-cadherin must be replenished at the plasma membrane by internal pools.

As an independent assay for internalization, we performed FRAP on the internalized Ecad-GFP in Scrb depleted cells. Under conditions blocking de novo protein synthesis (cycloheximide addition), a partial recovery of perinuclear GFP fluorescence occurred after bleaching, within 30 min at 37°C (Fig. S1). Since Ecad-GFP synthesis had been blocked, the perinuclear fluorescence must represent protein that had been retro-trafficked from the cortex.

### Internalized E-cadherin is Transported to the Golgi in Scrb-depleted Cells

We next sought to identify the membrane compartment into which E-cadherin accumulates after Scrb depletion. Partial colocalization was observed with Rab11, a marker of recycling endosomes, and with Golgin-97, which marks the trans-Golgi network (TGN) and late endosomes (Fig. S2). Partial overlap was also seen with the mannose-6-phosphate receptor, which recycles through the TGN [33] (Fig. 3A). However, the Golgi markers GM130 and Golgi 58K showed almost complete colocalization (Figs. 3B–C). Numb, an endocytic adapter protein thought to be involved in E-cadherin trafficking [34], remained tightly associated with the internalized cadherin (Fig. S2C). In addition, the perinuclear organization of the internalized E-cadherin was disrupted by addition of brefeldinA, which causes disintegration of the Golgi apparatus (Fig. S2D). We conclude, therefore, that loss of Scrb results, unexpectedly, in the endocytosis and retrieval of E-cadherin to the Golgi apparatus.

### Scrb Depletion Promotes the Binding of E-cadherin to Retromer Components

Cargo is commonly retrieved from the endosomal compartment to the Golgi via retromer complexes, which consist of various members of the sorting nexin (Snx) family plus a trimer of Vps26, Vps29, and Vps35 [25]. Consistent with a role for retromer in E-cadherin trafficking to the Golgi, silencing of Vps29 significantly reduced E-cadherin accumulation in the Golgi of Scrb-depleted cells (Fig. 4A–C), and E-cadherin was instead trapped in small vesicles scattered throughout the cytoplasm. A similar distribution was observed in WT cells depleted of Vps29 (Fig. 4A), possibly caused by a block in recycling to the plasma membrane. Consistent with a role for retromer in recruitment of E-cadherin to the Golgi, the amount of E-cadherin in association with endogenous Vps29 was low in control cells but was significantly increased by Scrb depletion (Fig. 4D). In addition, the recruitment of added rr1 antibody to perinuclear vesicles was reduced by depletion of Vps29 (Fig. S3). Taken together, these data support a role for retromer in the diversion of E-cadherin to the Golgi in Scrb-depleted cells.

### The E-cadherin/p120catenin Interaction is Disrupted in Cells Lacking Scrb

Does internalization of E-cadherin in Scrb-depleted cells result from a loss of junctional stability? Uncoupling of E-cadherin and p120 in mammalian epithelial cells disrupts adhesion and causes E-cadherin to be rapidly internalized and degraded [22], [35]. Therefore, we examined the interaction between these two proteins. Co-immunoprecipitations showed that the E-cadherin/p120 interaction is significantly diminished by loss of Scrb (Fig. 5A). The level of co-precipitated p120catenin from Scrb-depleted cells was reduced as compared to the control, although total p120catenin levels remained unchanged (Fig. 5B). The dissociation of p120 from E-cadherin was specific, because similar experiments to examine the E-cadherin/β-catenin complex showed no change in binding with loss of Scrb (Fig. 5C).

Moreover, although E-cadherin and p120catenin in control cells colocalize almost completely, silencing of Scrb caused a significant redistribution of p120 into the cytoplasm, as assessed by quantification of the overlap coefficient [36] and by linescans (Fig. 5D). These data are consistent with a partial disassociation from E-cadherin. By contrast, β-catenin remained strongly co-localized with E-cadherin in Scrb-depleted cells, on intracellular structures as well as at the disorganized lateral membrane, and was not diffusely distributed through the cytoplasm (Fig. 5D). Additionally, other cadherin binding proteins such as α-catenin and γ-catenin also remained co-distributed with E-cadherin in Scrb-depleted cells (Fig. S4A), indicating that the loss of p120/E-cadherin binding in these cells is highly specific. To test whether the disassociation of p120 caused by Scrb depletion might drive the internalization of E-cadherin, we reduced p120 levels by RNAi in cells expressing Ecad-GFP. As expected under these conditions [22], the amount of E-cadherin is greatly reduced (Fig. S4B, C); however, when the image brightness is increased, a fraction of the remaining Ecad-GFP can be detected in intracellular vesicles, though not in the same perinuclear pattern as occurs following depletion of Scrb (Fig. S4C). Loss of Scrb did not cause any detectable changes in the activities of known downstream effectors of p120-mediated signaling, such as the small GTPase Rap1 (Fig. S4D). Taken together, these data support a model in which Scrb normally stabilizes the association of p120 with E-cadherin, which reduces the dynamics of E-cadherin at the junctions and prevents endocytosis.

### Scrb is Required for Targeting of Internalized E-cadherin to the Lysosomes

Loss of p120 has been shown previously to result in the rapid degradation of internalized E-cadherin, mediated both by the proteosome and by transport to lysosomes [22]. Why then would the consequences of a reduced association of p120 with E-cadherin, caused by Scrb depletion, be different, leading to Golgi retrieval, instead of degradation? To examine this problem, we first confirmed by fluorescence imaging of Ecad-GFP and by immunoblotting of the endogenous protein that silencing of p120 results in the loss of E-cadherin (Fig. S4B, C). Next, to test whether the protein is targeted to lysosomes in p120-depleted cells we added leupeptin, a potent inhibitor of lysosomal proteases, for 4 h prior to imaging, then added Lysotracker for 10 min and imaged the live cells. As shown in Fig. S5, little colocalization was detected in control cells between Ecad-GFP and Lysotracker in the absence of leupeptin, but was clearly visible in cells lacking p120catenin. GFP accumulation in lysosomes was enhanced substantially by leupeptin and was higher in the p120-depleted cells. These observations are consistent with targeting of E-cadherin to the lysosomes in wild type cells, a process that is increased by loss of p120.

An important conclusion from our study is that Scrb not only helps to maintain E-cadherin at the cell cortex by promoting interaction with p120, but also plays a key role in blocking retromer binding and retrieval of E-cadherin to the Golgi, ensuring its degradation in lysosomes. A strong prediction from this model is that depletion of Scrb should over-ride the effect of silencing p120 and rescue E-cadherin expression, by switching traffic from the lysosomal pathway to Golgi retrieval. We tested this prediction by performing double knockdowns. As assessed by immunoblot (Fig. 6A) endogenous E-cadherin levels were not altered by Scrb depletion, were reduced by p120catenin depletion, but were efficiently rescued when both proteins were silenced. Silencing of Scrb did not alter the efficiency of knockdown by the shRNA against p120 (Fig. 6A). Immunofluorescence staining was consistent with the immunoblot data (Fig. 6 B): Ecad-GFP fluorescence was dramatically reduced by p120 depletion, but co-depletion of Scrb rescued Ecad-GFP expression and targeted the fusion protein to the Golgi. Note that the cortical Ecad-GFP fluorescence was also brighter in the double knockdown than in cells depleted of p120catenin alone, suggesting that the retrieved cadherin can be recycled back to the membrane even in the absence of p120.

## Discussion

The function of Scrb in mammalian cells has remained somewhat mysterious. Although essential for apical/basal polarity in *Drosophila* epithelia [10] it does not appear to perform a similar function in vertebrates. Instead, it is needed for planar cell polarity, perhaps through interaction with the Vangl tetraspanin proteins [12]. There is also some evidence of an involvement in vesicle traffic. Scrb binds to the C-terminus of the TSH receptor and over-expression of Scrb can inhibit TSHR endocytosis, through a βPIX-GIT1-ARF6 pathway [9]. We have found that Scrb is also required for intercellular adhesion through E-cadherin, but through a mechanism independent of βPIX [17]. In addition, we found no evidence for any change in ARF6 activity following depletion of Scrb in MDCK cells (data not shown). We propose that Scrb promotes p120 association with E-cadherin, which stabilizes E-cadherin at the lateral cortex, (Fig. 5C). Reciprocally, expression of E-cadherin is necessary for the localization of Scrb to the adherens junction, presumably through p120 [37]. In our earlier study we had not observed intracellular E-cadherin in Scrb-depleted cells, and cell surface biotinylation did not detect any reduction in surface-exposed E-cadherin [17], but using more efficient Scrb depletion and improved immunofluorescence assays, unequivocally identified internalized E-cadherin retrieval to the Golgi apparatus. The reason that no significant decrease in biotinylated E-cadherin was detected in our previous study might be a consequence of differences in accessibility of the protein between wild type cells, where junctions are stable, versus Scrb-depleted cells in which intercellular adhesion is reduced. Trans-dimerization between E-cadherin molecules on adjacent cells will hold the plasma membranes sufficiently close together that diffusion of the biotinylation reagent is likely to be reduced, so that labeling would be sub-stoichiometric. This effect would be substantially less in Scrb-depleted cells, so labeling would be more efficient, even though there is less E-cadherin available to be labeled, resulting in a similar total level of labeling as in the wild type cells.

In terms of mechanism, we have discovered that Scrb blocks the association of E-cadherin with a retromer complex containing Vps29, preventing retrieval of the protein to the Golgi apparatus. A consequence of this blockage is that any endocytosed E-cadherin is normally sorted to the lysosomes for degradation rather than being recycled. Importantly, targeting to either compartment is independent of p120 binding to E-cadherin, because the cadherin can enter either pathway in the absence of p120: the lysosomal pathway in the presence of Scrb, or the retrieval pathway in the absence of Scrb. Therefore, the association of Scrb with p120 must be independent of Scrb’s ability to block retrieval (Fig. 5C). Scrb might either block recruitment of retromer, or it might help recruit factors that sort E-cadherin to the lysosomes.

Our data provide evidence that a polarity protein can select the itinerary for internalized membrane proteins, and the first indication that a polarity protein regulates the retromer pathway. In *Drosophila* ectoderm the loss of Par-6, Par-3 or Cdc42 can cause arrest of endocytosed apical proteins or of E-cadherin in early endosomes [1], [2], [38], and in *C. elegans* the polarity proteins are required for efficient endocytosis and recycling [4]; but these studies did not identify a switch in cargo trafficking from one destination to another. The Crumbs polarity protein is a known cargo for the retromer complex, and depends on retromer for recycling to the apical membrane [39]. However, it remains unknown if endocytosed Crumbs passes through the Golgi before being recycled. Important questions for the future are whether mammalian Scrb regulates sorting of other cargo in addition to E-cadherin and the TSHR, and whether Scrb acts to interfere sterically with retromer recognition of E-cadherin, or alters the type of sorting nexin that associates with the endocytosed protein. It will also be interesting to know whether this new function of Scrb plays any role in planar cell polarity.

## Supporting Information

Figure S1 **Effects of Scrb depletion on lateral membranes and E-cadherin.** (A) Control and Scrb-depleted cells cells were plated on slides for 2 d then fixed and stained for Na/K ATPase and GFP. GFP was used as a transfection marker. (B) FRAP of Ecad-GFP in MDCK cells. Cells nucleofected with shRNA against Scrb were incubated for 3 d then placed at 37°C on the stage of a Zeiss LSM510 Meta confocal microscope. Areas of interest were drawn within individual cells to encircle the Golgi and were bleached using 3 iterations at 100% transmission, 75% laser power with the 25 watt argon laser. Cells were imaged at intervals after bleaching using 3% transmission. Two sets of representative images are shown, pre-bleach, bleach and 30 min post-bleach.
(PDF)

Figure S2 **Localization of internalized Ecadherin.** Control and Scrb-depleted Ecad-GFP cells were grown on slides for 48 h, fixed and immunostained for (A) Golgin-97, or (B) Rab11. (C) Cells were stained for endogenous E-cadherin and Numb. (D) Cells expressing Ecad-GFP were nucleofected with shRNAs as shown then treated with 1 mM brefeldin dissolved in DMSO, to a final concentration of 10 uM; or with an equivalent amount of DMSO alone as a negative control, for 2 h, and imaged for Ecad-GFP distribution. All images are confocal sections (Zeiss LSM 510; 40× oil immersion lens, NA 1.4).
(PDF)

Figure S3 **Silencing of Vps29 blocks Golgi accumulation of endocytosed E-cadherin.** Cells were incubated for 1 hr at 4°C with the RR1 antibody against the extracellular domain of E-cadherin. They were then washed to remove free RR1, and either left at 4°C or switched to 37°C for 4 h. The cells were then fixed, permeabilized and stained for total E-cadherin and for the RR1 antibody. White arrows show the accumulation of E-cadherin and RR1 in perinuclear regions in cells depleted of Scrb alone. All images are confocal sections (Zeiss LSM 510; 40× oil immersion lens, NA 1.4).
(PDF)

Figure S4 **Internalized E-cadherin remains associated with α- and γ-catenin.** (A) Cells expressing Ecad-GFP were transfected with shRNA vectors as shown (LucKD as a control, or ScrbKD to silence Scrb expression) and after 3d they were fixed and stained for α-catenin or γ-catenin and imaged by confocal microscopy. Representative line scans are shown. In addition, 5 fields per condition were quantified for overlap of the red and green channels and overlap coefficients were calculated using Openlab software. No significant differences were observed between the control and Scrb-depleted cells. (B) Immunoblot of lysates from cells nucleofected with shRNAs targeting Scrb or p120catenin, showing destruction of E-cadherin is promoted by loss of p120catenin but not Scrb. (C) Localization and expression of Ecad-GFP in cells nucleofected as in (B). (D) Control and Scrb-depleted cells were incubated in calcium-free medium overnight, then switched to calcium-containing medium and lysed at indicated times after calcium re-addition. Active Rap1GTPase was precipitated onto glutathione-Sepharose beads bound to GST Ral1-GDS. Samples were analyzed by immunoblot for Rap1 to detect Rap1-GTP. Band intensities were quantified using ImageJ and normalized to total Rap1 in the lysates.
(PDF)

Figure S5 **Depletion of p120catenin drives E-cadherin to the lysosomes.** MDCK cells expressing Ecad-GFP were nucleofected with control or p120catenin-targeted shRNAs, and grown on chambered coverslips. After 3 d some chambers were treated with 100 µg/ml of leupeptin to inhibit lysosomal proteases. After 2 h, red Lysotracker Red DND-99 was added to a final concentration of 100 nM. The cells were then imaged live, at 37°C, by confocal microscopy. Overlap coefficients were calculated using Openlab software. Boxes on right provide a 3× magnification of the regions outlined in white on the merged images. The green channel was enhanced using Photoshop Levels to stretch the contrast from 0 ->255 to the values shown.
(PDF)

## References

[1] Georgiou M, Marinari E, Burden J, Baum B (2008) Cdc42, Par6, and aPKC regulate Arp2/3-mediated endocytosis to control local adherens junction stability. Curr Biol 18: 1631–1638. 18976918 10.1016/j.cub.2008.09.029

[2] Harris KP, Tepass U (2008) Cdc42 and Par proteins stabilize dynamic adherens junctions in the Drosophila neuroectoderm through regulation of apical endocytosis. J Cell Biol 183: 1129–1143. 19064670 10.1083/jcb.200807020 PMC2600741

[3] Bryant DM, Datta A, Rodriguez-Fraticelli AE, Peranen J, Martin-Belmonte F, et al (2010) A molecular network for de novo generation of the apical surface and lumen. Nat Cell Biol 12: 1035–1045. 20890297 10.1038/ncb2106 PMC2975675

[4] Balklava Z, Pant S, Fares H, Grant BD (2007) Genome-wide analysis identifies a general requirement for polarity proteins in endocytic traffic. Nat Cell Biol 9: 1066–1073. 17704769 10.1038/ncb1627

[5] Lalli G (2009) RalA and the exocyst complex influence neuronal polarity through PAR-3 and aPKC. J Cell Sci 122: 1499–1506. 19383721 10.1242/jcs.044339

[6] Musch A, Cohen D, Yeaman C, Nelson WJ, Rodriguez-Boulan E, et al (2002) Mammalian homolog of Drosophila tumor suppressor lethal (2) giant larvae interacts with basolateral exocytic machinery in Madin-Darby canine kidney cells. Mol Biol Cell 13: 158–168. 11809830 10.1091/mbc.01-10-0496 PMC65098

[7] Moreau MM, Piguel N, Papouin T, Koehl M, Durand CM, et al (2010) The planar polarity protein Scribble1 is essential for neuronal plasticity and brain function. J Neurosci 30: 9738–9752. 20660256 10.1523/JNEUROSCI.6007-09.2010 PMC6632828

[8] Sun Y, Aiga M, Yoshida E, Humbert PO, Bamji SX (2009) Scribble interacts with beta-catenin to localize synaptic vesicles to synapses. Mol Biol Cell 20: 3390–3400. 19458197 10.1091/mbc.E08-12-1172 PMC2710836

[9] Lahuna O, Quellari M, Achard C, Nola S, Meduri G, et al (2005) Thyrotropin receptor trafficking relies on the hScrib-betaPIX-GIT1-ARF6 pathway. Embo J 24: 1364–1374. 15775968 10.1038/sj.emboj.7600616 PMC1142541

[10] Bilder D, Perrimon N (2000) Localization of apical epithelial determinants by the basolateral PDZ protein Scribble. Nature 403: 676–680. 10688207 10.1038/35001108

[11] Pagliarini RA, Xu T (2003) A genetic screen in Drosophila for metastatic behavior. Science 302: 1227–1231. 14551319 10.1126/science.1088474

[12] Murdoch JN, Henderson DJ, Doudney K, Gaston-Massuet C, Phillips HM, et al (2003) Disruption of scribble (Scrb1) causes severe neural tube defects in the circletail mouse. Hum Mol Genet 12: 87–98. 12499390 10.1093/hmg/ddg014

[13] Albertson R, Chabu C, Sheehan A, Doe CQ (2004) Scribble protein domain mapping reveals a multistep localization mechanism and domains necessary for establishing cortical polarity. J Cell Sci 117: 6061–6070. 15536119 10.1242/jcs.01525

[14] Zeitler J, Hsu CP, Dionne H, Bilder D (2004) Domains controlling cell polarity and proliferation in the Drosophila tumor suppressor Scribble. J Cell Biol 167: 1137–1146. 15611336 10.1083/jcb.200407158 PMC2172630

[15] Audebert S, Navarro C, Nourry C, Chasserot-Golaz S, Lecine P, et al (2004) Mammalian Scribble forms a tight complex with the betaPIX exchange factor. Curr Biol 14: 987–995. 15182672 10.1016/j.cub.2004.05.051

[16] Kallay LM, McNickle A, Brennwald PJ, Hubbard AL, Braiterman LT (2006) Scribble associates with two polarity proteins, Lgl2 and Vangl2, via distinct molecular domains. J Cell Biochem 99: 647–664. 16791850 10.1002/jcb.20992

[17] Qin Y, Capaldo C, Gumbiner BM, Macara IG (2005) The mammalian Scribble polarity protein regulates epithelial cell adhesion and migration through E-cadherin. J Cell Biol 171: 1061–1071. 16344308 10.1083/jcb.200506094 PMC2171311

[18] Brieher WM, Yap AS, Gumbiner BM (1996) Lateral dimerization is required for the homophilic binding activity of C-cadherin. J Cell Biol 135: 487–496. 8896604 10.1083/jcb.135.2.487 PMC2121050

[19] Reynolds AB, Daniel J, McCrea PD, Wheelock MJ, Wu J, et al (1994) Identification of a new catenin: the tyrosine kinase substrate p120cas associates with E-cadherin complexes. Mol Cell Biol 14: 8333–8342. 7526156 10.1128/mcb.14.12.8333-8342.1994 PMC359372

[20] Yap AS, Niessen CM, Gumbiner BM (1998) The juxtamembrane region of the cadherin cytoplasmic tail supports lateral clustering, adhesive strengthening, and interaction with p120ctn. J Cell Biol 141: 779–789. 9566976 10.1083/jcb.141.3.779 PMC2132752

[21] Huber AH, Weis WI (2001) The structure of the beta-catenin/E-cadherin complex and the molecular basis of diverse ligand recognition by beta-catenin. Cell 105: 391–402. 11348595 10.1016/s0092-8674(01)00330-0

[22] Davis MA, Ireton RC, Reynolds AB (2003) A core function for p120-catenin in cadherin turnover. J Cell Biol 163: 525–534. 14610055 10.1083/jcb.200307111 PMC2173649

[23] Bryant DM, Kerr MC, Hammond LA, Joseph SR, Mostov KE, et al (2007) EGF induces macropinocytosis and SNX1-modulated recycling of E-cadherin. J Cell Sci 120: 1818–1828. 17502486 10.1242/jcs.000653

[24] Carlton J, Bujny M, Rutherford A, Cullen P (2005) Sorting nexins–unifying trends and new perspectives. Traffic 6: 75–82. 15634208 10.1111/j.1600-0854.2005.00260.x

[25] Bonifacino JS, Hurley JH (2008) Retromer. Curr Opin Cell Biol 20: 427–436. 18472259 10.1016/j.ceb.2008.03.009 PMC2833274

[26] O'Brien LE, Jou TS, Pollack AL, Zhang Q, Hansen SH, et al (2001) Rac1 orientates epithelial apical polarity through effects on basolateral laminin assembly. Nat Cell Biol 3: 831–838. 11533663 10.1038/ncb0901-831

[27] Adams CL, Chen YT, Smith SJ, Nelson WJ (1998) Mechanisms of epithelial cell-cell adhesion and cell compaction revealed by high-resolution tracking of E-cadherin-green fluorescent protein. J Cell Biol 142: 1105–1119. 9722621 10.1083/jcb.142.4.1105 PMC2132880

[28] Chen X, Macara IG (2006) RNA interference techniques to study epithelial cell adhesion and polarity. Methods Enzymol 406: 362–374. 16472670 10.1016/S0076-6879(06)06026-5

[29] Dorfman J, Macara IG (2008) STRADalpha regulates LKB1 localization by blocking access to importin-alpha, and by association with Crm1 and exportin-7. Mol Biol Cell 19: 1614–1626. 18256292 10.1091/mbc.E07-05-0454 PMC2291406

[30] Asuri S, Yan J, Paranavitana NC, Quilliam LA (2008) E-cadherin dis-engagement activates the Rap1 GTPase. J Cell Biochem 105: 1027–1037. 18767072 10.1002/jcb.21902 PMC2657844

[31] Davis MA, Reynolds AB (2006) Blocked acinar development, E-cadherin reduction, and intraepithelial neoplasia upon ablation of p120-catenin in the mouse salivary gland. Dev Cell 10: 21–31. 16399075 10.1016/j.devcel.2005.12.004

[32] Wang Q, Chen XW, Margolis B (2007) PALS1 regulates E-cadherin trafficking in mammalian epithelial cells. Mol Biol Cell 18: 874–885. 17182851 10.1091/mbc.E06-07-0651 PMC1805083

[33] Lin SX, Mallet WG, Huang AY, Maxfield FR (2004) Endocytosed cation-independent mannose 6-phosphate receptor traffics via the endocytic recycling compartment en route to the trans-Golgi network and a subpopulation of late endosomes. Mol Biol Cell 15: 721–733. 14595110 10.1091/mbc.E03-07-0497 PMC329388

[34] Wang Z, Sandiford S, Wu C, Li SS (2009) Numb regulates cell-cell adhesion and polarity in response to tyrosine kinase signalling. Embo J 28: 2360–2373. 19609305 10.1038/emboj.2009.190 PMC2712596

[35] Thoreson MA, Anastasiadis PZ, Daniel JM, Ireton RC, Wheelock MJ, et al (2000) Selective uncoupling of p120(ctn) from E-cadherin disrupts strong adhesion. J Cell Biol 148: 189–202. 10629228 10.1083/jcb.148.1.189 PMC2156209

[36] Manders EMM, Verbeek FJ, Aten JA (1993) Measurement of co-localization of objects in dual-colour confocal images. Journal of Microscopy 169: 375–382. 10.1111/j.1365-2818.1993.tb03313.x 33930978

[37] Navarro C, Nola S, Audebert S, Santoni MJ, Arsanto JP, et al.. (2005) Junctional recruitment of mammalian Scribble relies on E-cadherin engagement. Oncogene. 10.1038/sj.onc.1208632 15806148

[38] Leibfried A, Fricke R, Morgan MJ, Bogdan S, Bellaiche Y (2008) Drosophila Cip4 and WASp define a branch of the Cdc42-Par6-aPKC pathway regulating E-cadherin endocytosis. Curr Biol 18: 1639–1648. 18976911 10.1016/j.cub.2008.09.063

[39] Pocha SM, Wassmer T, Niehage C, Hoflack B, Knust E (2011) Retromer controls epithelial cell polarity by trafficking the apical determinant crumbs. Curr Biol 21: 1111–1117. 21700461 10.1016/j.cub.2011.05.007

