# Supplementary figures and images for: The Scribble Polarity Protein Stabilizes E-Cadherin/p120-Catenin Binding and Blocks Retrieval of E-Cadherin to the Golgi
Source: PLoS One. 2012 Nov 30;7(11):e51130. doi: 10.1371/journal.pone.0051130 (PMC3511384; doi:10.1371/journal.pone.0051130)

Fig. S1 Lohia et al

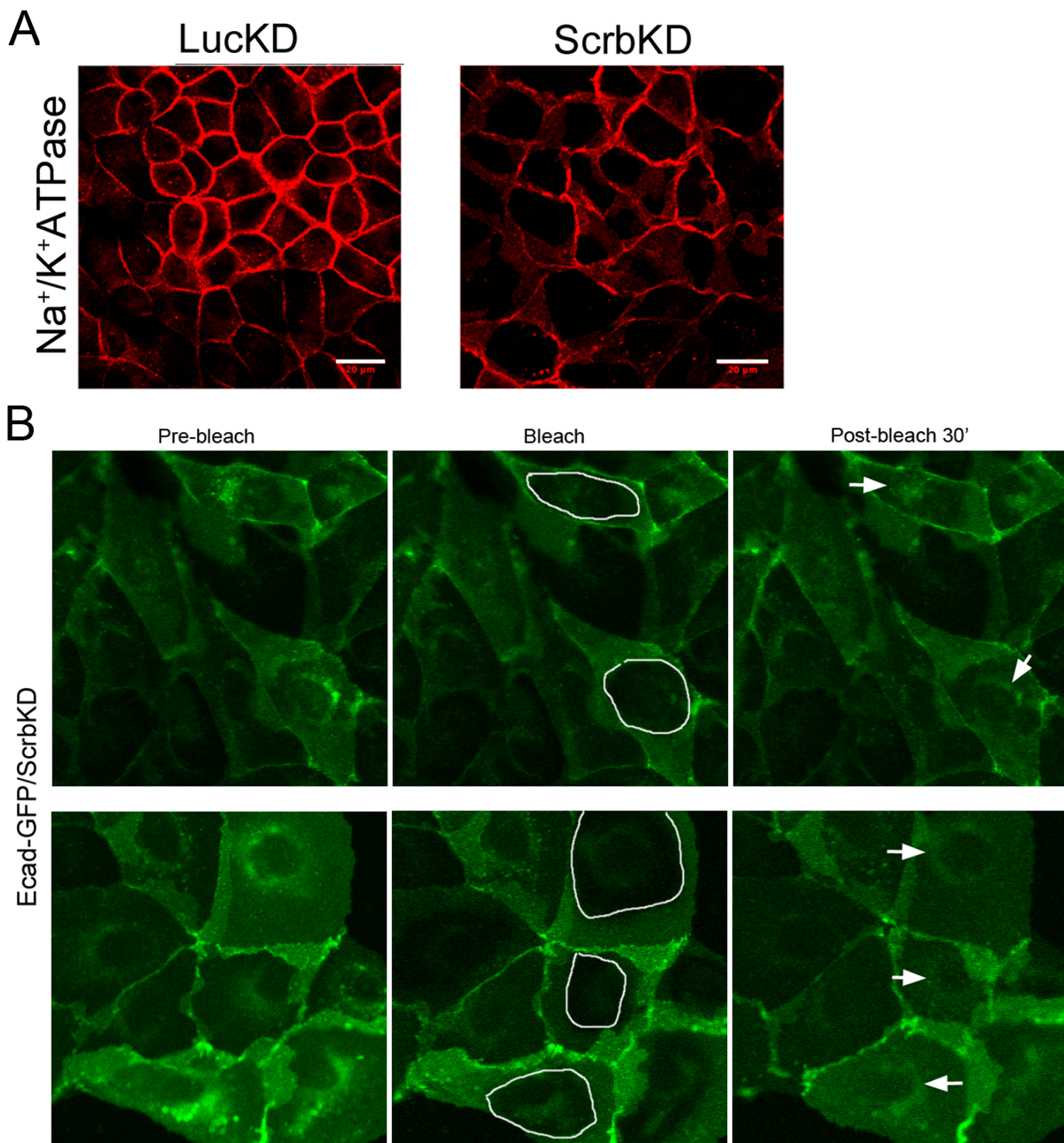

Supplement: Figure S1 — Effects of Scrb depletion on lateral membranes and E-cadherin. (A) Control and Scrb-depleted cells cells were plated on slides for 2 d then fixed and stained for Na/K ATPase and GFP. GFP was used as a transfection marker. (B) FRAP of Ecad-GFP in MDCK cells. Cells nucleofected with shRNA against Scrb were incubated for 3 d then placed at 37°C on the stage of a Zeiss LSM510 Meta confocal microscope. Areas of interest were drawn within individual cells to encircle the Golgi and were bleached using 3 iterations at 100% transmission, 75% laser power with the 25 watt argon laser. Cells were imaged at intervals after bleaching using 3% transmission. Two sets of representative images are shown, pre-bleach, bleach and 30 min post-bleach. (PDF) [file pone.0051130.s001.pdf]

Fig. S2 Lohia et al

A

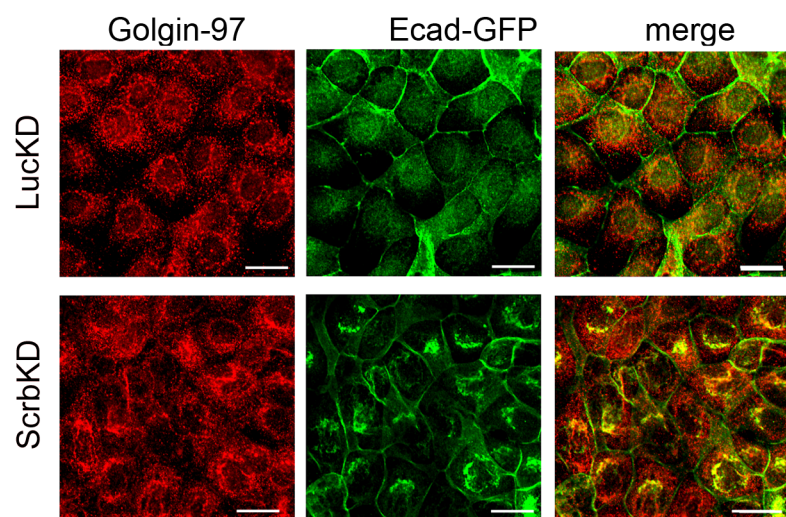

B

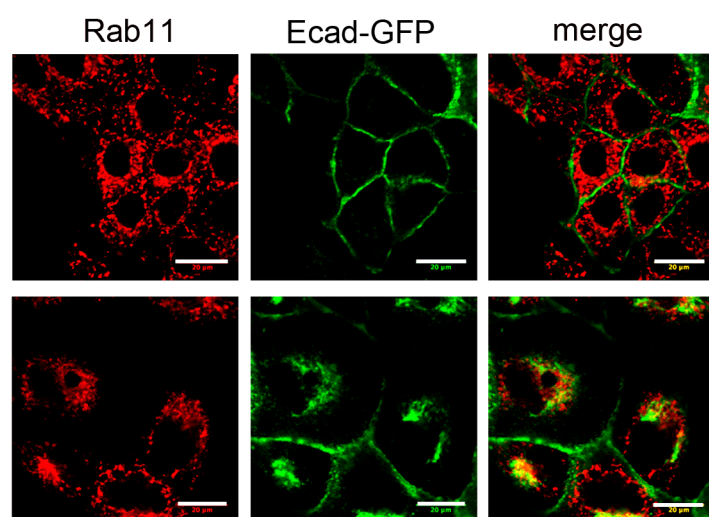

C

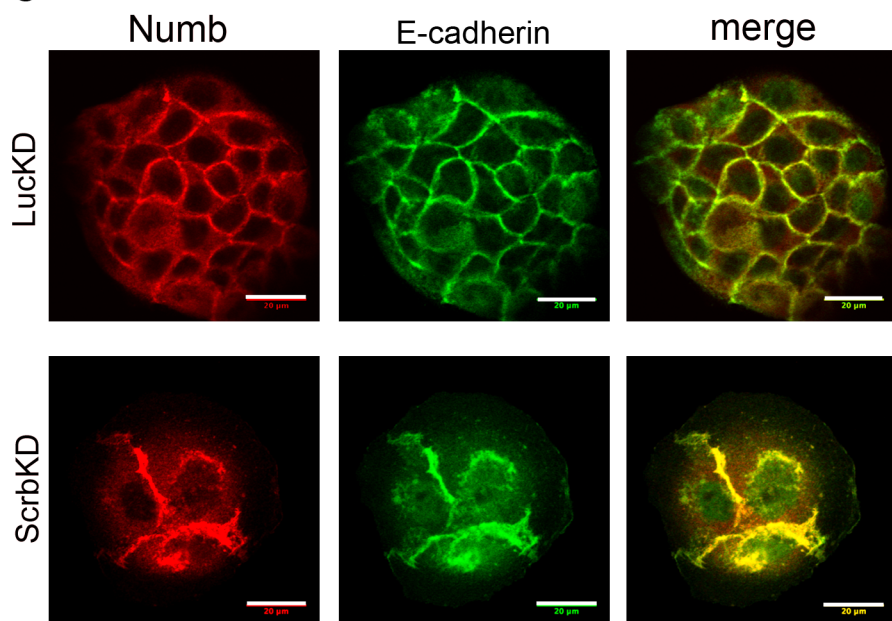

D

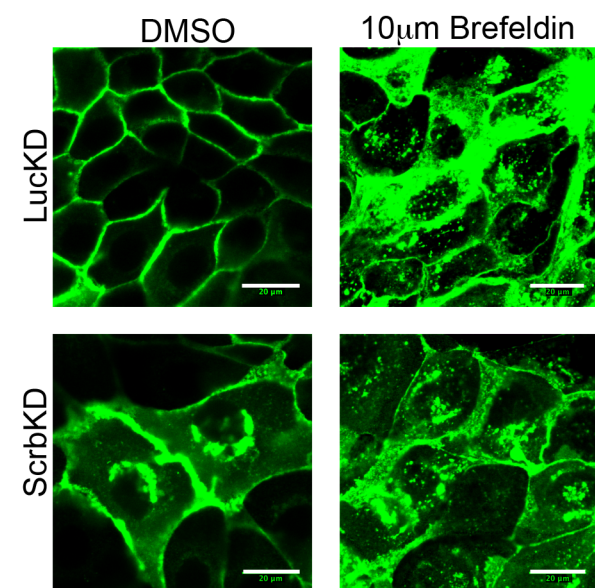

Supplement: Figure S2 — Localization of internalized Ecadherin. Control and Scrb-depleted Ecad-GFP cells were grown on slides for 48 h, fixed and immunostained for (A) Golgin-97, or (B) Rab11. (C) Cells were stained for endogenous E-cadherin and Numb. (D) Cells expressing Ecad-GFP were nucleofected with shRNAs as shown then treated with 1 mM brefeldin dissolved in DMSO, to a final concentration of 10 uM; or with an equivalent amount of DMSO alone as a negative control, for 2 h, and imaged for Ecad-GFP distribution. All images are confocal sections (Zeiss LSM 510; 40× oil immersion lens, NA 1.4). (PDF) [file pone.0051130.s002.pdf]

Fig. S3 Lohia et al

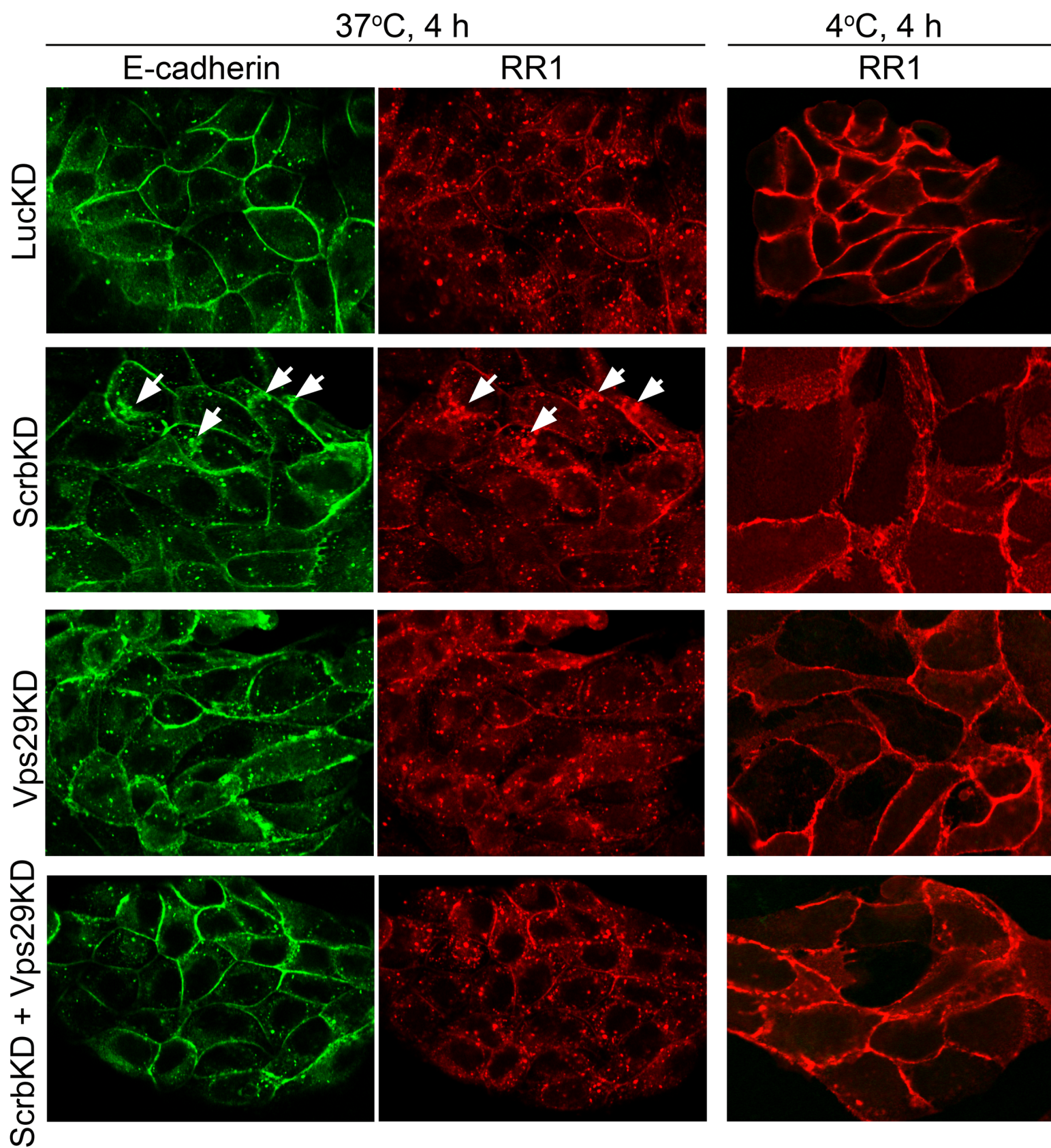

Supplement: Figure S3 — Silencing of Vps29 blocks Golgi accumulation of endocytosed E-cadherin. Cells were incubated for 1 hr at 4°C with the RR1 antibody against the extracellular domain of E-cadherin. They were then washed to remove free RR1, and either left at 4°C or switched to 37°C for 4 h. The cells were then fixed, permeabilized and stained for total E-cadherin and for the RR1 antibody. White arrows show the accumulation of E-cadherin and RR1 in perinuclear regions in cells depleted of Scrb alone. All images are confocal sections (Zeiss LSM 510; 40× oil immersion lens, NA 1.4). (PDF) [file pone.0051130.s003.pdf]

Fig.S4 Lohia et al

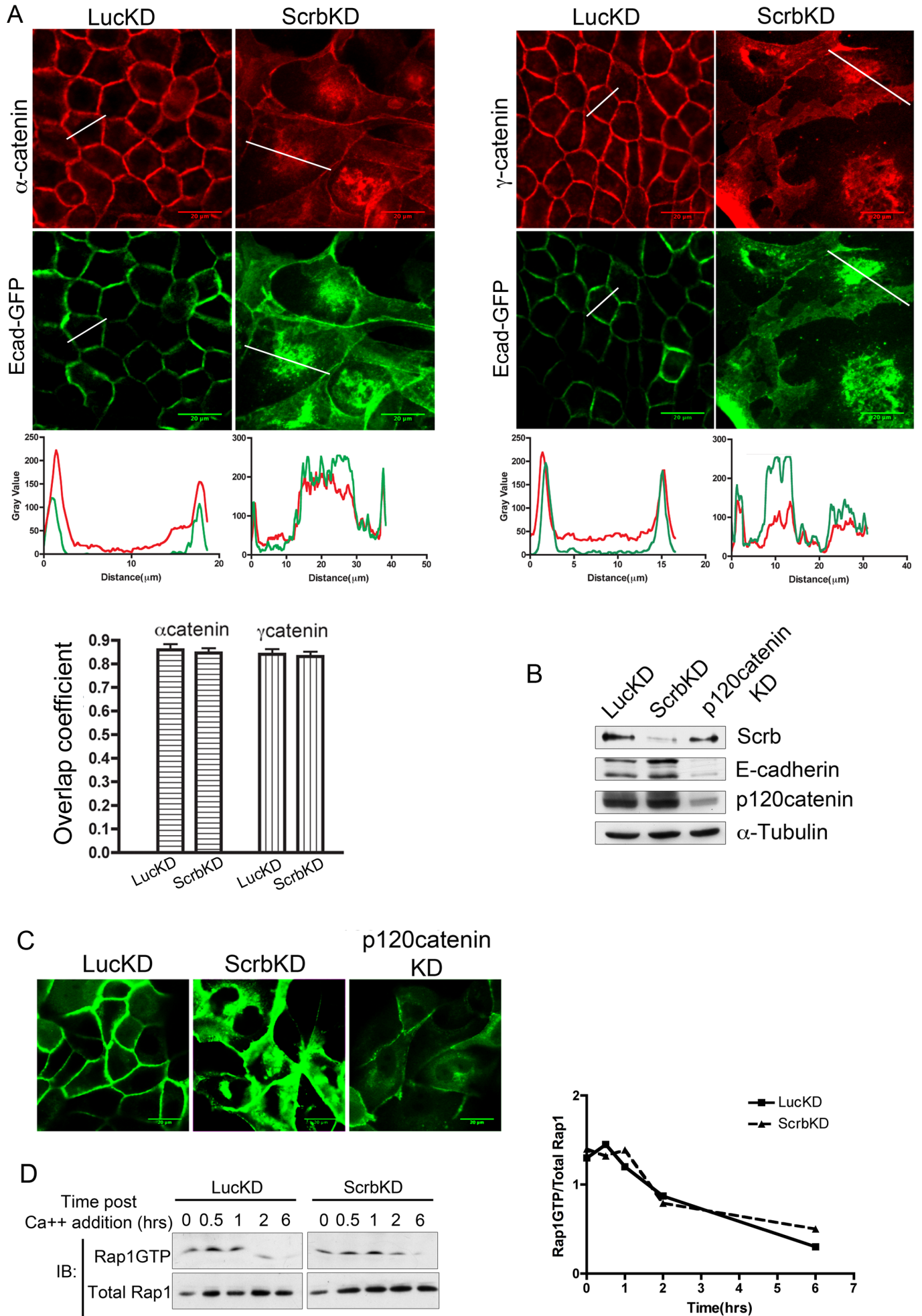

Supplement: Figure S4 — Internalized E-cadherin remains associated with α- and γ-catenin. (A) Cells expressing Ecad-GFP were transfected with shRNA vectors as shown (LucKD as a control, or ScrbKD to silence Scrb expression) and after 3d they were fixed and stained for α-catenin or γ-catenin and imaged by confocal microscopy. Representative line scans are shown. In addition, 5 fields per condition were quantified for overlap of the red and green channels and overlap coefficients were calculated using Openlab software. No significant differences were observed between the control and Scrb-depleted cells. (B) Immunoblot of lysates from cells nucleofected with shRNAs targeting Scrb or p120catenin, showing destruction of E-cadherin is promoted by loss of p120catenin but not Scrb. (C) Localization and expression of Ecad-GFP in cells nucleofected as in (B). (D) Control and Scrb-depleted cells were incubated in calcium-free medium overnight, then switched to calcium-containing medium and lysed at indicated times after calcium re-addition. Active Rap1GTPase was precipitated onto glutathione-Sepharose beads bound to GST Ral1-GDS. Samples were analyzed by immunoblot for Rap1 to detect Rap1-GTP. Band intensities were quantified using ImageJ and normalized to total Rap1 in the lysates. (PDF) [file pone.0051130.s004.pdf]

Fig. S5 Lohia et al

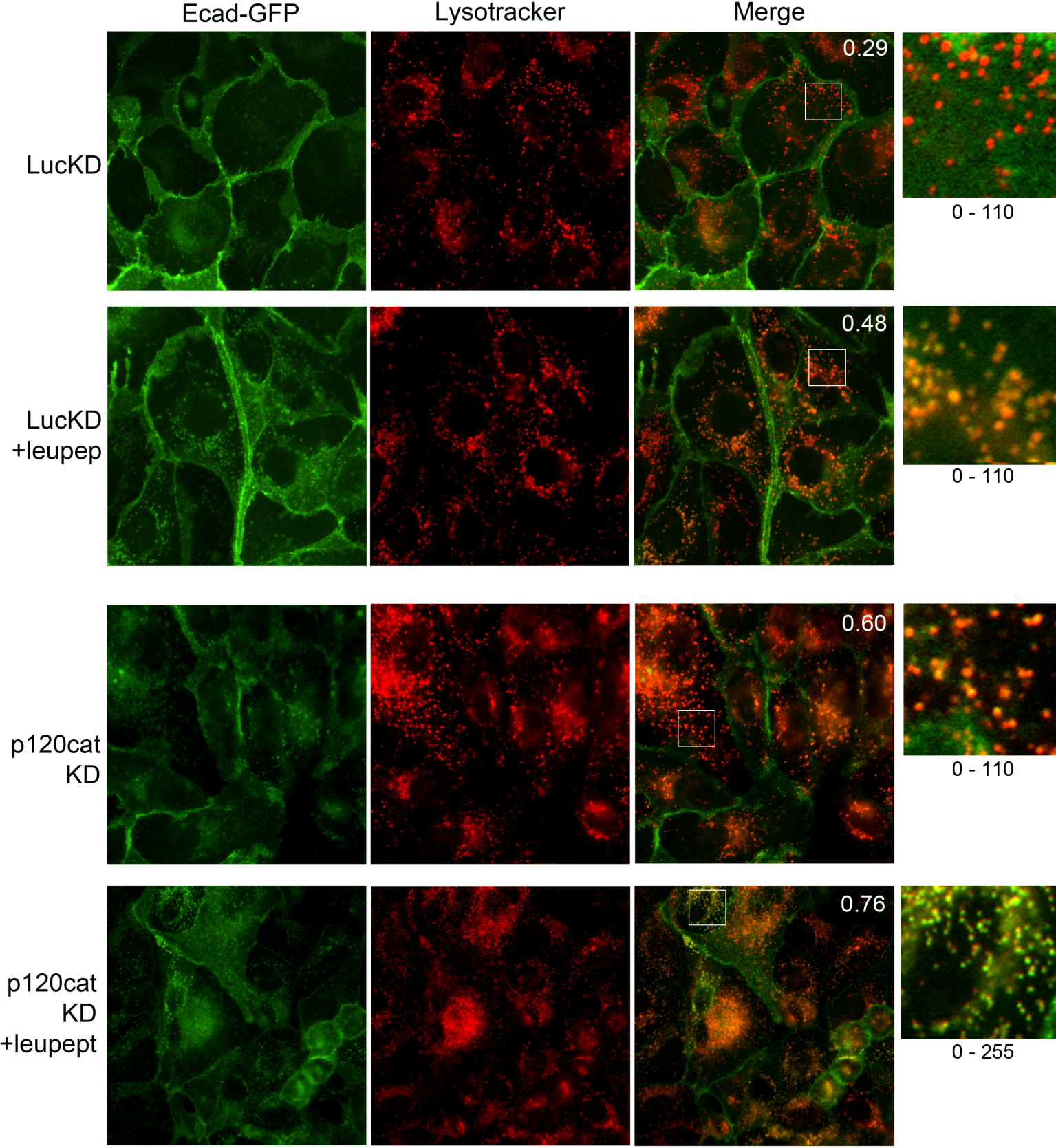

Supplement: Figure S5 — Depletion of p120catenin drives E-cadherin to the lysosomes. MDCK cells expressing Ecad-GFP were nucleofected with control or p120catenin-targeted shRNAs, and grown on chambered coverslips. After 3 d some chambers were treated with 100 µg/ml of leupeptin to inhibit lysosomal proteases. After 2 h, red Lysotracker Red DND-99 was added to a final concentration of 100 nM. The cells were then imaged live, at 37°C, by confocal microscopy. Overlap coefficients were calculated using Openlab software. Boxes on right provide a 3× magnification of the regions outlined in white on the merged images. The green channel was enhanced using Photoshop Levels to stretch the contrast from 0 ->255 to the values shown. (PDF) [file pone.0051130.s005.pdf]
